# DNA self-assembly nanoflower reverse P-glycoprotein mediated drug resistance in chronic myelogenous leukemia therapy

**DOI:** 10.3389/fbioe.2023.1265199

**Published:** 2023-08-21

**Authors:** Pengxuan Zhao, Yeteng Zhong, Pengcheng Pan, Shasha Zhang, Yu Tian, Jun Zhang, Guohui Yi, Zhendong Zhao, Tiantian Wu

**Affiliations:** ^1^ Key Laboratory of Tropical Translational Medicine of Ministry of Education, Hainan Provincial Key Laboratory for Research and Development of Tropical Herbs, Haikou Key Laboratory of Li Nationality Medicine, School of Pharmacy, Hainan Medical University, Haikou, China; ^2^ Department of Clinical Laboratory, The Second Affiliated Hospital, Hainan Medical University, Haikou, China; ^3^ Wuhan Wuchang Hospital, Wuchang Hospital Affiliated to Wuhan University of Science and Technology, Wuhan, China; ^4^ Public Research Center Hainan, Hainan Medical University, Haikou, China; ^5^ Analytical and Testing Center of Hainan University, Hainan University, Haikou, China; ^6^ Jiangsu Hengrui Pharmaceuticals Co., Ltd., Lianyungang, China; ^7^ Department of Medical Ultrasound, Tongji Hospital, Tongji Medical College, Huazhong University of Science and Technology, Wuhan, China

**Keywords:** DNA self-assembly, drug resistance, drug delivery, chronic myelogenous leukemia, rolling circle amplification

## Abstract

**Introduction:** Chronic myelogenous leukemia (CML) is a clonal myeloproliferative disorder caused by the BCR-ABL chimeric tyrosine kinase. Vincristine (VCR) is widely used in leukemia therapy but is hindered by multidrug resistance (MDR).

**Methods:** We prepared DNA nanoflower via self-assembly for the delivery of VCR and P-glycoprotein small interfering RNA (P-gp siRNA).

**Results and Discussion:** The as-prepared nanoflower had a floriform shape with high loading efficiency of VCR (80%). Furthermore, the nanoflower could deliver VCR and P-gp siRNA into MDR CML cells and induce potent cytotoxicity both *in vitro* and *in vivo*, thus overcoming MDR of CML. Overall, this nanoflower is a promising tool for resistant CML therapy.

## 1 Introduction

Chronic myelogenous leukemia (CML), also called as chronic myeloid leukemia, is a kind of clonal hematopoietic stem cell disease (Kantarjian et al., 1988; Cortes et al., 1996; Faderl et al., 1999a; Faderl et al., 1999b; Egan and Radich, 2017). CML is caused by the formation of oncogenic BCR-ABL gene fusion and accounts for nearly 15% of the adult leukemias (Kalidas et al., 2001; Jin et al., 2016; Bellavia et al., 2017; Zhang et al., 2018). Vincristine (VCR) is a derivate of Madagascan periwinkle, which could bind to tubulin and disrupt microtubules, thus inhibiting cell division (Johnson et al., 1963; Moncrief and Lipscomb, 1965; Rosenthal and Kaufman, 1974). VCR as an efficient chemotherapeutic drug, has been widely used for various tumor therapy including leukemia (Tsuruo et al., 1981; Buckner et al., 2016; Mora et al., 2016; Hawkins et al., 2018). However, like other chemotherapy, VCR is prone to induce the emergence of multidrug resistance (MDR), which could over-express the P-glycoprotein (P-gp) (Feng et al., 2020; Wang et al., 2021). Thereby, chemotherapeutic agents would be outflowed from the cancer cells and leading to the failure of chemical therapy.

To overcome the resistance problem, small interfering RNA (siRNA) has been extensively used for MDR gene silencing (Meng et al., 2013; Shen et al., 2014; Yhee et al., 2015; Subhan and Torchilin, 2019; Wang et al., 2019). For example, Wang et al. and Liu et al. separately prepared mesoporous silica or polymer nanoparticles to co-deliver P-gp siRNA and doxorubicin for overcoming drug resistance (Yuan et al., 2021; Liu et al., 2022). Hence, it is believed that the combination of VCR and P-gp siRNA might be useful for reversing the drug resistance of chronic myelogenous leukemia.

**FIGURE 1:**
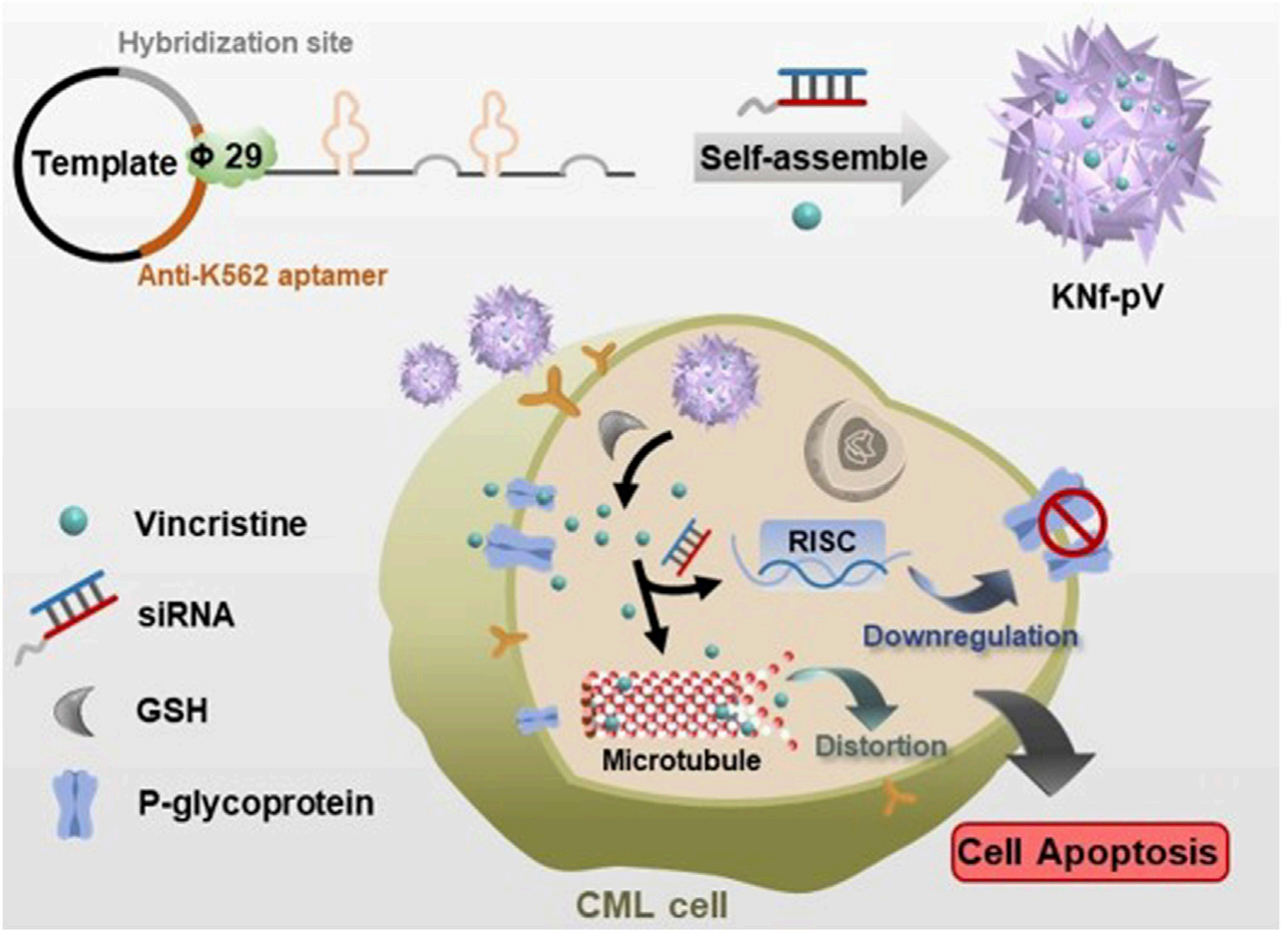
Schematic illustration of the self-assembly nanoflower for the reverse of drug resistance in chronic myelogenous leukemia treatment.

Recently, oligonucleotide-based probes, also known as aptamers, have been widely used due to the affinity and specificity (Tuerk and Gold, 1990; Keefe et al., 2010). Like antibodies, aptamers could specifically recognize a variety of objectives from small molecules to cells (Shangguan et al., 2006; Geiger et al., 1996). Moreover, aptamers have more advantages compared with antibodies, such as better permeability, no immunogenicity, and easy to chemical synthesis (Xiong et al., 2013). Thus in this study, we designed a self-assembly based delivery platform modified with aptamer for synergistic therapy in drug resistant CML (Figure 1). The drug-loaded nanoflower, namely, KNf-pV, was decorated with CML cell K562-specific aptamer to allow selective recognition and enhanced internalization of tumor cells. Chemical therapy drug VCR and gene silencing drug siRNA (siP-gp) were loaded by interaction and hybridization with rolling circle amplification (RCA)-produced DNA strand. The siP-gp delivered by drug-loaded delivery system (KNf-pV) with stimuli-responsive linker could significantly inhibit the expression of drug resistant-related P-gp, which consequently enhanced the chemosensitivity of CML cells in cancer therapy and reverse the drug resistance. Using the VCR-siRNA co-loaded nanoflower, we realized the reversal of drug resistance and synergistic cancer therapy both *in vitro* and *in vivo*. This delivery platform provides a promising strategy for resistant cancer therapy.

## 2 Materials and methods

### 2.1 Materials

All the oligonucleotides in this work were obtained from Huzhou Hippo Biotechnology Co., Ltd. (Huzhou, China), and the oligonucleotides used in this work are listed in Supplementary Table S1. T4 DNA ligase and Phi29 DNA polymerase were purchased from New England BioLabs (Beverly, MA, United States). Dulbecco’s modified Eagle’s medium (DMEM), and fetal bovine serum were purchased from Gibco. Cell culture dishes/plates, round coverslips, and centrifuge tubes were obtained from NEST Biotechnology Co. Ltd. (Wuxi, China). Hoechst 33,342 was purchased from Abbkine Scientific (Wuhan, China). Calcein-AM staining kit was purchased from Solarbio kit (Beijing, China). Annexin V-fluorescein-5-isothiocyanate (AV-FITC)/PI double staining kit was purchased from Elabscience Biotechnology Co., Ltd.

### 2.2 One-pot synthesis and characterization of the KNf-pV

The phosphorylated linear ssDNA (0.6 μM) and primers (1.2 μM) were annealed to form a circular DNA template. T4 DNA ligase (5 U/μL) was added and incubated at 16°C for 8 h. Then, the circular template was incubated with Phi29 DNA polymerase (1 U/μL), dNTP (2 mM), and reaction buffer for 8 h at 37°C to synthesis the nanoflower. For the preparation of the drug-loaded KNf-pV, different ratio of VCR and siP-gp was added in the reaction mixture. Then the NFs were diluted in the ultrapure water and centrifuged to get the precipitate. The morphologies of NFs were determined using scanning electron microscopy (SEM, JEOL, JSM-7500F) and atomic force microscope (AFM, MultiMode 8, Bruker).

### 2.3 Relative drug release efficiency

The co-loaded nanoflower was dispersed in PBS buffer with or without adding GSH (5 mM). The mixture was kept at 37°C with continuous shaking. The buffer containing released VCR was separated by Amicon stirred cell at different time points for quantification. The concentration of VCR was analyzed by high performance liquid chromatography (HPLC, SPD-20A, Shimadzu, Kyoto, Japan) with ultraviolet detection at 298 nm (Ling et al., 2010; Xu and Qiu, 2015). Chromatographic separation was carried out on a C18 column (250 mm × 4.6 mm, 5 μm) using methanol-ammonium acetate (5 mM)-acetic acid (60:40:0.1, V/V/V) as mobile phase.

### 2.4 Cellar uptake

To analyze the uptake of nanoflower *in vitro*. K562 and K562/VCR cells were seeded in 24-well plates at 1 × 10^5^ cells per well and incubated with nanoflower for 4 h. The cells were then washed with PBS for three times and fixed in 4% para-formaldehyde for 15 min. Lysosome was stained with Lysotracker, Nuclei were counterstained with 4’,6-diamidino-2-phenylindole (DAPI). The cells were imaged under a confocal laser scanning microscopy (CLSM, IX81; Olympus, Tokyo, Japan).

### 2.5 MCSs

Multicellular tumor spheroid (Friedrich et al., 2009) was rendered for testing the internalization of nanoflower. The K562/vcr cells were seeded and cultured overnight. The T75 flask was pre-covered by 10 mL of hot agarose (1 w/v %) and cooled to completely solidified. 10^6^ cells were seeded in a flask and incubated for 72 h. The MCSs were treated with NF-PV (without aptamer) or KNf-PV (with aptamer). The drug concentration was 100 μg/mL based on VCR. After 4 h of incubation at 37°C, the spheroids were collected, washed with PBS for three times, and stained by Calcein-AM for 1 h at room temperature. The spheroids were fixed with PFA 4% (w/v) in PBS for 1 h at room temperature and observed with confocal laser scanning microscopy (LSM 710 CLSM, Carl Zeiss, Jena, Germany).

### 2.6 *In vitro* cytotoxicity

Cell viability was determined by Annexin V/PI staining and MTT assay. For Annexin V/PI staining, K562 and K562/VCR cells were seeded in 24-well plates at 1 × 10^5^ cells per well for overnight. After 24 h of treatment with free VCR, Nf-pV, or KNf-pV. Cells were further stained with Annexin V and propidium iodide (PI) for 10 min. Subsequently, cells were washed with PBS and analyzed using a confocal laser scanning microscope. For MTT assay, K562 and K562/VCR cells were seeded in 96-well plates at 2 × 10^4^ cells per well and cultured overnight. After 24 h of treatment with Nf, free VCR, Nf-pV, KNf-V, or KNf-pV, MTT solution was added. After an additional 4 h incubation, the supernatants were removed carefully and followed by the addition of 150 μL per well of DMSO. Absorbance was measured at 570 nm using the SpectraMax M5 microplate reader.

### 2.7 qRT-PCR

Total RNA was extracted from the cells using Trizol reagent following the protocol suggested by the manufacturer. Then, cDNA was synthesized using the Reverse Transcription System (Promega, Madison, WI, United States). Real-time PCR was performed with SYBR Green probe on a Mx3005PQPCR instrument from Agilent Technologies. rRNA was used as the input reference.

### 2.8 Western blotting

Cell lysates were resolved on 12% SDS-PAGE and incubated with antibody, developed by an enhanced chemiluminescence detection kit from Thermo Fisher Scientific (Waltham, MA, United States). Antibodies used for Western blotting including those against P-gp (1:1,000, Abcam) and β-actin (1:1,000, Abcam).

### 2.9 *In vivo* anti-tumor effect

BALB/c nude (female, 5–6 weeks, SPF level) with tumors that were xenografted by injecting 5 × 10^6^ cells per mouse in the upper right blanks. When tumor volume reached 60 mm^3^, PBS, free VCR, or KNf-pV were administrated by tail vein injection (0.5 mg/mL VCR) on day 0, 3, and 6. Mice were weighted regularly. At 18 days after tumor inoculation, tumors were collected and weighed.

## 3 Results and discussion

### 3.1 Construction of the KNf-pV

**FIGURE 2:**
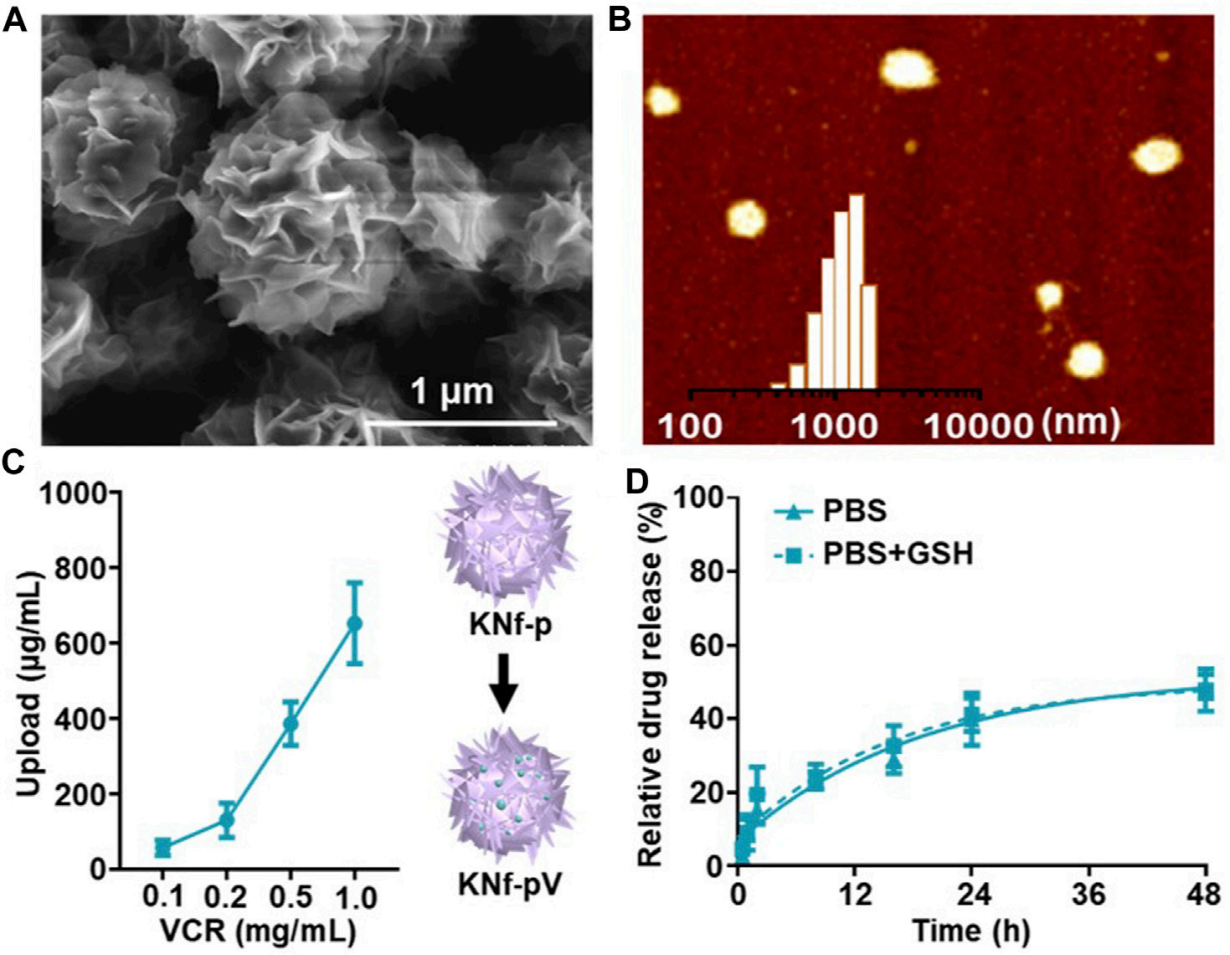
**(A)** SEM images of KNf-pV. Scale bar: 1 μm. **(B)** AFM image and size distribution of KNf-pV. **(C)** VCR loading efficacy analysis. **(D)** Release of VCR under different conditions.

The programmability of DNA nanoflowers enables the incorporation of functional DNA moieties into nanoflowers through rational design of the DNA templates. In this study, anti-K562 aptamer (KK1B10) was chosen for CML recognized (Yang et al., 2011; Ge et al., 2015). VCR was chosen for CML therapy. To generate DNA nanoflower by the rolling circle amplification, phosphorylated linear templates were circularized using DNA ligase. Efficient production of DNA leads to an increased local DNA concentration and resulted in the construction of the drug-loaded delivery system, namely, KNf-Pv. Scanning electron microscope (SEM) observed that KNf-pV were floriform in shape with a size of about 1 μm (Figure 2A), which was similar to the images of atomic force microscopy (AFM, Figure 2B). In addition, the loading efficiency of VCR in KNf-pV was nearly 80% (Figure 2C). The release of VCR from KNf-pV was analyzed by dialysis. As shown in Figure 2D, KNf-pV showed obviously sustained-release property in PBS solution, and almost half of VCR was released in 48 h. The siRNA loaded in the delivery system was released after the addition of GSH (Supplementary Figure S1). Excellent release performance is crucial for nanoparitcles as drug delivery system used for cancer treatment (Wu et al., 2019; Zhu et al., 2023a; Zhu et al., 2023b; Zhu et al., 2023c).

### 3.2 Cellular uptake of cells and multicellular tumor spheroids (MCSs)

**FIGURE 3:**
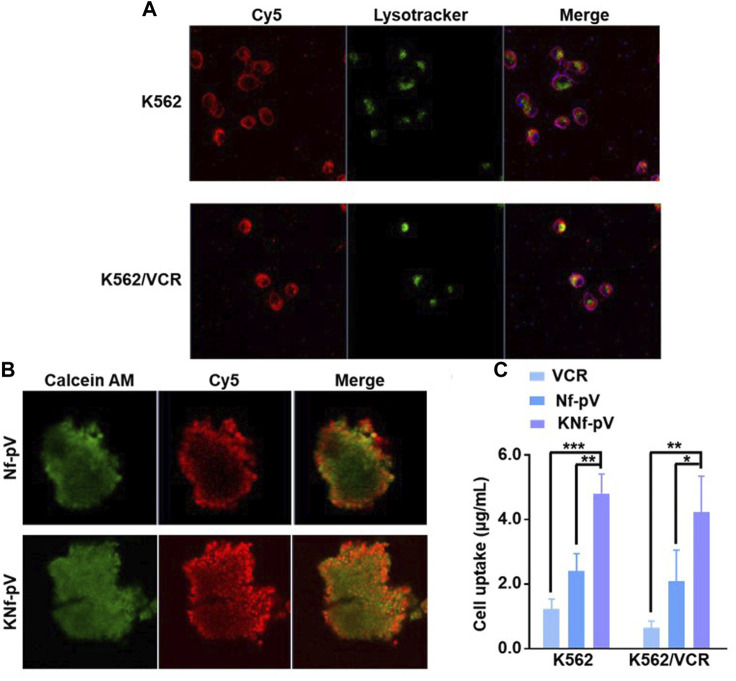
**(A)** Confocal images of K562 and K562/VCR cells treated with KNf-pV. Nanoflower was labeled with Cy5, red; Lysosome was stained with Lysotracker, green **(B)** Confocal images of K562/VCR MCSs. Cells were stained with Calcein-AM, green; Nanoflower was labeled with Cy5, red. **(C)** VCR accumulation analysis in K562 and K562/VCR MCSs.

To test whether KNf-pV could enter into CML cells. Cellular uptake was performed. K562 cells or drug-resistant K562/VCR cells were treated with Cy5-labeled KNf-pV, and lysosome was stained with Lysotracker. As shown in Figure 3A, the fluorescence of KNf-pV was not co-localized with lysosome, and distributed throughout the cytoplasm of both K562 and K562/VCR cells. These results demonstrated the lysosome escape of KNf-pV. The confocal images of multicellular tumor spheroids (MCSs) of K562/VCR also showed enhanced accumulation and permeability of KNf-pV (Figure 3B).

To further demonstrate VCR uptake in K562 and K562/VCR cells, the *in vitro* quantitative accumulation study was performed. Nf-pV group increased the cell uptake of VCR compared with free VCR group both in K562 and K562/VCR cells. After aptamer decoration, the VCR uptake was further improved in KNf-pV group (4.8 μg/mL in K562 cells, 4.2 μg/mL in K562/VCR cells, Figure 3C). Moreover, similar results were observed in normalized data (Supplementary Figure S2). These data suggested that KNf-pV can realize lysosome escape and deliver VCR in both K562 and K562/VCR cells.

### 3.3 *In vitro* cytotoxicity analysis

**FIGURE 4:**
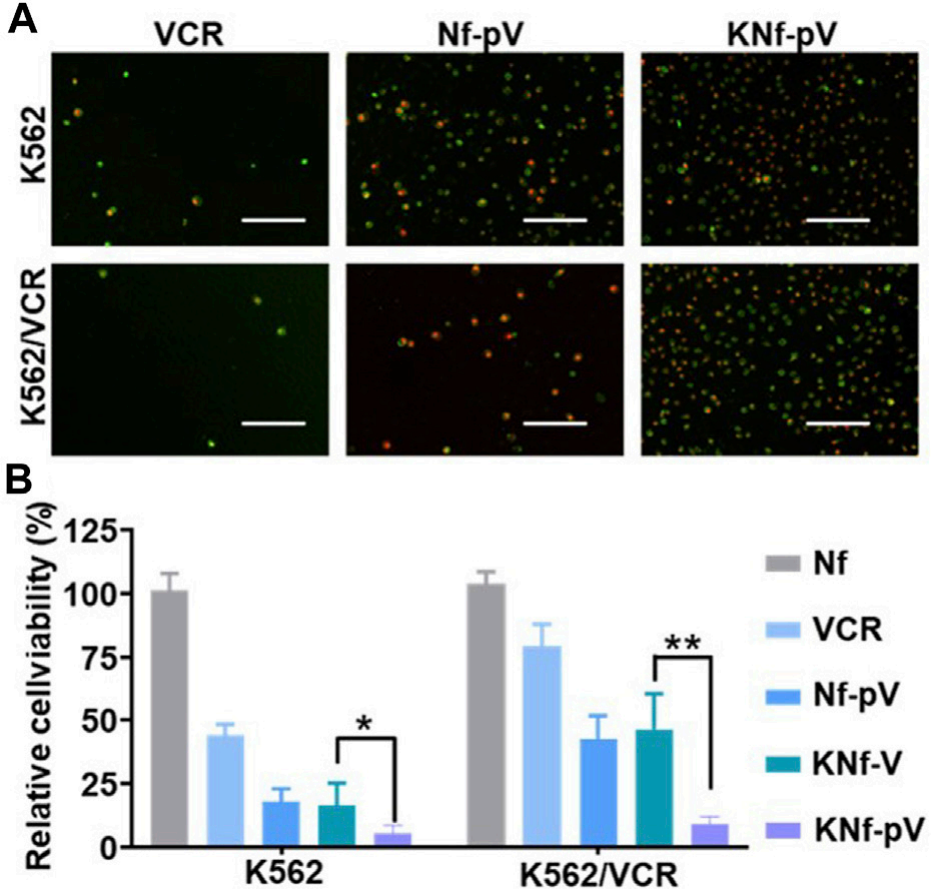
**(A)** Cell apoptosis imaging. Cells were stained with Annexin V-FITC and PI after being cultured with drugs. **(B)** Cell viability analysis of cells after the indicated treatments. (**p < 0.01).

To test the cell death mechanism, the annexin V/propidium iodide (PI) assay was performed in K562 and K562/VCR cells (Figure 4A). When the cells were treated with free VCR, moderate changes in the morphology and fluorescent signals from annexin V/PI were observed, especially in K562/VCR cells, which demonstrated that free VCR did not cause considerable damage to K562 and K562/VCR cells. Whereas, the fluorescent signals were significantly enhanced in Nf-pV group, proving that the cell membrane was damaged. Furthermore, the fluorescent signals of KNf-pV group were obviously stronger than any other groups, indicating the most powerful cytotoxicity.

Next, the *in vitro* cytotoxicity was further carried out through methyl thiazolyl tetrazolium (MTT) assay. As expected (Figure 4B), moderate cytotoxic effect was observed in free VCR group, more than 75% of the K562/VCR cells were alive. In contrast, Nf-pV group and KNf-V group exhibited increased cytotoxic effect, which killed nearly 50% of the K562/VCR cells. Notably, KNf-pV group showed the strongest cytotoxicity (only 12% of the K562/VCR cells was alive), indicating the synergistic effect of siP-gp and VCR after aptamer modified. Overall, these data clearly proved that the KNf-pV could induce cytotoxicity in K562 and K562/VCR cells.

### 3.4 The drug resistance reversal of KNf-pV

**FIGURE 5:**
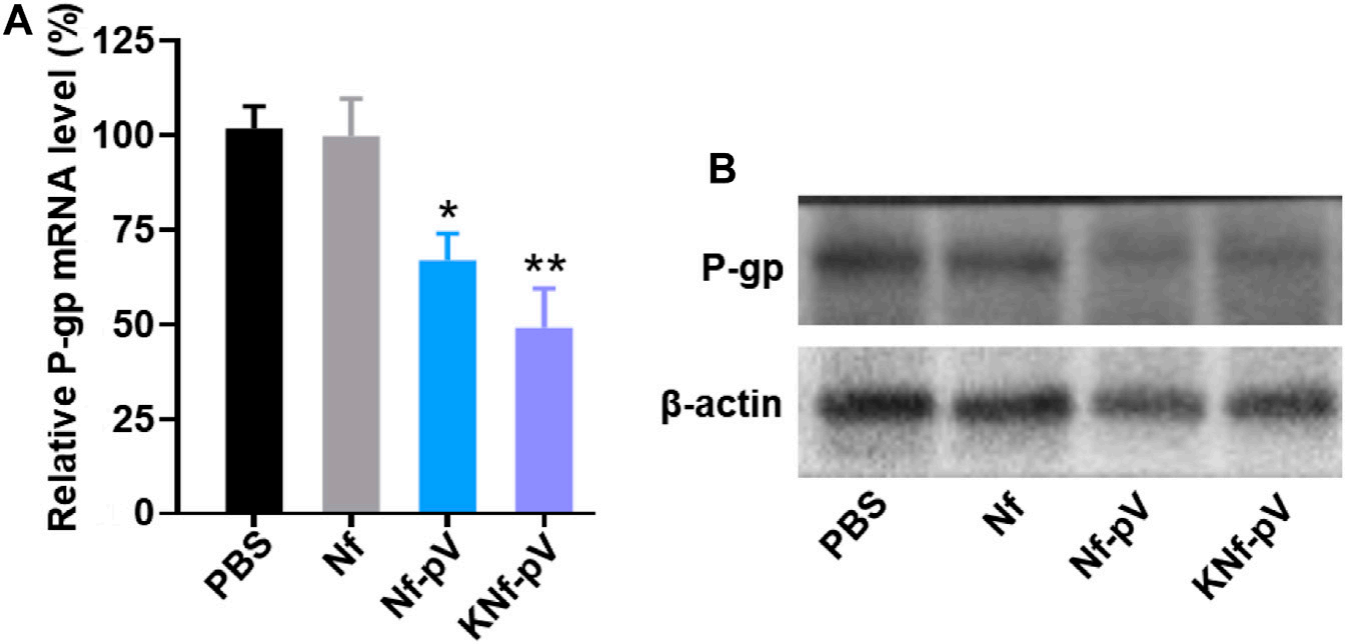
**(A)** Quantitative RT-PCR analysis of relative P-gp mRNA level to β-actin (an internal control). **(B)** Western blots detection of the expression level of P-gp.

To study the drug resistance reversal effect of KNf-pV, we measured the expression of P-gp by quantitative RT-PCR and western blots. P-gp expression levels of KNf-pV group were significantly downregulated both in quantitative RT-PCR and western blots results (Figures 5A, B).

### 3.5 Tumor inhibitory effect *in vivo*

**FIGURE 6:**
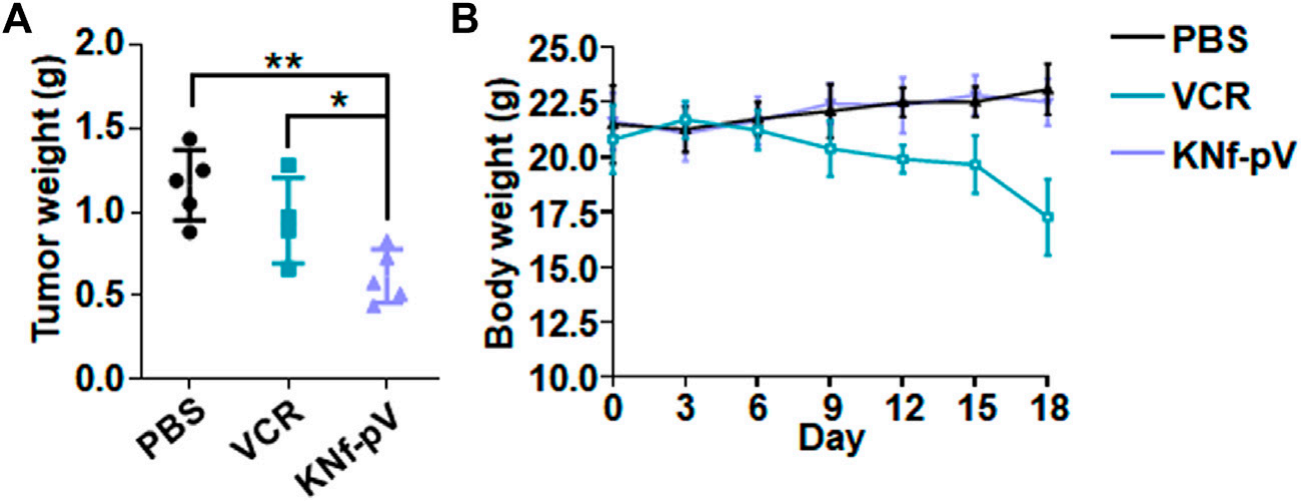
**(A)** Tumor weight of each group. **(B)** Body weight changes of each group during the therapy.

Encouraged by the excellent antitumor effects *in vitro* of KNf-pV, we evaluated the antitumor efficacy *in vivo* through a xenografted mouse model. When the subcutaneous tumor reached 60 mm^3^, three doses of PBS, free VCR, or KNf-pV were administrated through tail vein injection at 0.5 mg/mL VCR dose every 3 days. After 18 days, all mice were sacrificed and tumors were removed for imaging. Compared with PBS (0.8 g) group, free VCR group (0.7 g) showed lower tumor weight. Furthermore, KNf-pV group has the least tumor weight (0.4 g) without significantly reducing the body weight (Supplementary Figure S3; Figures 6A, B). By contrast, the body weight of free VCR group was markedly decreased due to the systemic toxicity of free VCR (Figure 6B). Altogether, these results indicated that the synergistic effect could be realized *in vivo* by KNf-pV.

## 4 Conclusion

In this study, we have developed a novel K562-specific aptamer decorated DNA nanoflower to realize the co-delivery of VCR and P-gp siRNA (KNf-pV) for overcoming drug resistance in CML therapy. DNA nanoflower was self-assembled from DNA during RCA, which could avoid the complicated sequence design and incorporate functionalities including aptamers for specific recognizing, and drug-binding DNA sequences for specific drug delivery. Specifically, the VCR loading efficiency of KNf-pV was as high as 80%. The KNf-pV could effectively enter into CML cells and induce therapeutic effect both *in vitro* and *in vivo*. Moreover, our KNf-pV could reduce the expression of P-gp thus overcoming the drug resistance of VCR. Taken together, our designed DNA nanoflower provides a platform for MDR cancer therapy.

## Data Availability

The original contributions presented in the study are included in the article/Supplementary Material, further inquiries can be directed to the corresponding authors.
