# Supplementary material for: DNA self-assembly nanoflower reverse P-glycoprotein mediated drug resistance in chronic myelogenous leukemia therapy
Source: Front Bioeng Biotechnol. 2023 Aug 21;11:1265199. doi: 10.3389/fbioe.2023.1265199 (PMC10475561; doi:10.3389/fbioe.2023.1265199)
Supplement: Supplementary file 1 [file DataSheet1.docx]

Supplementary Material

DNA self-assembly Nanoflower reverse P-glycoprotein mediated drug resistance in chronic myelogenous leukemia therapy

Pengxuan Zhao^1＃^, Yeteng Zhong,^2＃^ Pengcheng Pan^1,＃^ , Shasha Zhang^3^, Yu Tian^4^, Guohui Yi^5*^, Zhendong Zhao^6*^, Tiantian Wu^1*^

*** Correspondence:**

Guohui Yi

guohuiyi6@hainmc.edu.cn

Zhendong Zhao

[zhaozhendong@hainanu.edu.cn](mailto:zhaozhendong@hainanu.edu.cn)

Tiantian Wu

[hy0207149@hainmc.edu.cn](mailto:hy0207149@hainmc.edu.cn)

# Supplementary Figures


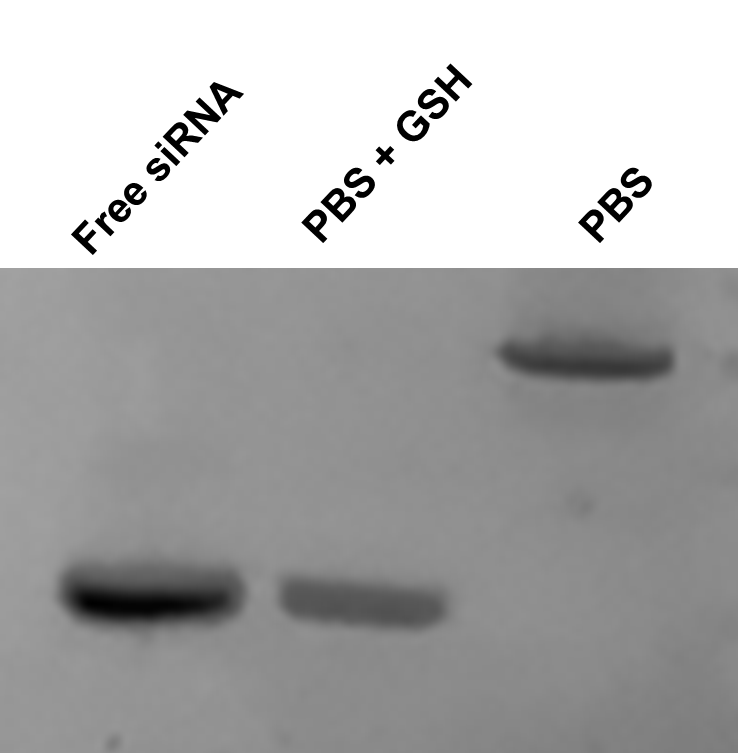
**Supplementary Figure 1.** Agarose gel analysis of KNf-pV after incubated with GSH for 24 h. The concentration of GSH added in PBS was 5 mM.


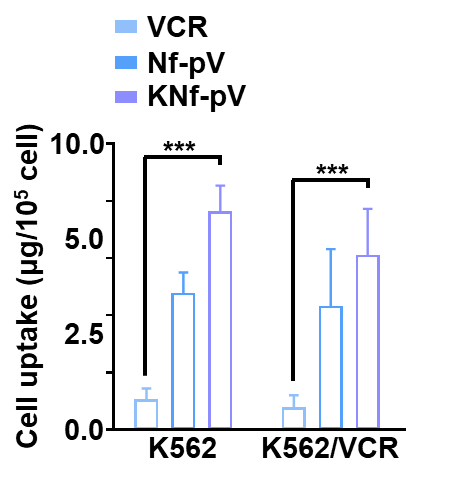


**Supplementary Figure 2.** VCR accumulation analysis in K562 and K562/VCR cells.


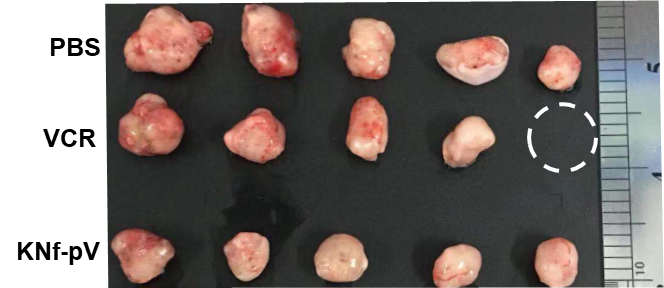


**Supplementary Figure 3.** Pictures of the excised tumors after the treatment.


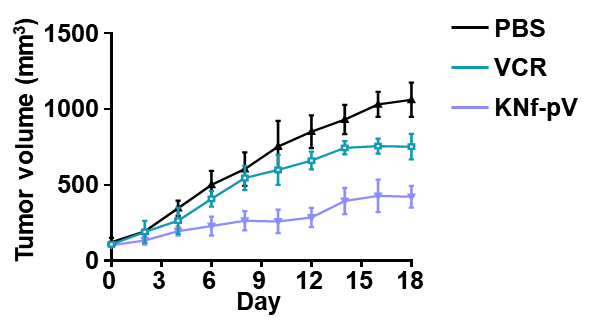


**Supplementary Figure 4.** Tumor volume changes of each group during the therapy.

**Table S1.** DNA sequences for Nanoflower.

| **Name** | **Sequence (5’-3’)** |
| --- | --- |
| **Template** | Phosphate-TCAGGAGAAGATAGACTGATCTGCTGTTGCTGCAGCACGCGTTTGTGCTACTCCAGTTCTTTACAGCTTCACCTATAAATAGGAACCCA |
| **siRNA** | CGGAAGGCCUAAUGCCGAAdTdT  GAACTGGAGTAGCACAAA-S-S-UUCGGCAUUAGGCCUUCCGdTdG |
